# Perioperative education for patients undergoing colorectal stoma surgery: A scoping review

**DOI:** 10.1016/j.ijnsa.2026.100604

**Published:** 2026-06-16

**Authors:** Marina Bogiatzis, Karen Gerrard, Wendy Smyth

**Affiliations:** a Townsville Institute of Health Research and Innovation, Townsville University Hospital, 100 Angus Smith Drive, Douglas, Queensland 4814, Australia; b Nursing and Midwifery, James Cook University, Townsville, Queensland 4811, Australia

**Keywords:** Perioperative, Patient education, Enterostomal therapy nursing, Colorectal, Enterostomy, Wound, ostomy and continence nursing

## Abstract

**Background:**

Colorectal ostomy surgery with stoma formation, presents significant physical, psychological, and social challenges for patients. Perioperative education is essential for supporting patients and facilitating adjustment to life with a stoma.

**Aim:**

To identify the education strategies for patients undergoing colorectal ostomy surgery and summarise the outcomes measured.

**Methods:**

Four databases (CINAHL, MEDLINE, PsycINFO and Scopus) were systematically searched for peer reviewed research articles up to mid-November 2024. Studies were included if they examined patient educational strategies and outcomes related to colorectal ostomy surgery.

**Findings:**

Thirty-five articles from 18 countries were included. Most interventions used face-to-face nurse-led education supplemented with written materials or multimedia tools such as telehealth and mobile applications. Patient involvement in intervention design emerged in recent studies and three studies included peer‑led education, leading to improved self‑efficacy, psychosocial adjustment, and satisfaction. Overall, education improved ostomy knowledge, self‑care skills and quality of life. Preoperative education was effective in reducing anxiety and depression, whilst postoperative education supported long‑term adaptation. Interventions combining preoperative and postoperative components were linked to shorter length of stay and fewer complications.

**Discussion:**

Comprehensive education and ongoing support from specialist nurses, family and peers improves patient outcomes. Multimedia tools show potential to extend education to widespread and diverse patient populations, complementing the benefits of face-to-face care.

**Conclusion:**

Stoma education has evolved toward multi-modal, personalised, and technology-enabled approaches delivered across the perioperative pathway. These integrated strategies have the greatest potential to improve patient outcomes and optimise health service utilisation.

**What is already known:**

• Limited reviews of ostomy education, with mixed results of associated outcomes, make it difficult to advise best practice.
• Colorectal surgery with ostomy formation has a high risk of complications and long-term lifestyle challenges for patients.
• Involvement of specialist nurses contributes to improved patient outcomes.

Alt-text: Unlabelled box dummy alt text

**What this paper adds:**

• This review identifies a shift toward multi-modal, technology-enabled, participatory education.
• Highlights personalised, continuous perioperative education improving outcomes.
• Identifies limited evidence on caregiver involvement and digital equity.

Alt-text: Unlabelled box dummy alt text

## 1 Introduction

Approximately 50,000 Australians are currently living with a colorectal or urological ostomy (Department of Health and Aged Care, 2024). Colorectal surgery resulting in an ostomy is required when the body “has lost normal bowel function due to disease, injury, a birth defect or other causes” (Haines, 2023, p.5). Such surgery results in a colostomy or ileostomy, depending on the segment of bowel that is diverted. While the aim of surgery is to help people return to their usual life activities, managing an intestinal ostomy (often called a stoma, particularly when referring to the physical opening) can bring significant physical, psychological, social challenges (Alenezi et al., 2021).

Ostomates (people with a stoma) and their carers frequently report reduced quality of life, anxiety, altered body image, and social isolation (Ayaz-Alkaya, 2019). Whilst evidence shows that effective education improves patient outcomes and reduces healthcare costs (Danielsen et al., 2013), the educational strategies vary across hospitals (Faury et al., 2017). Previous studies have often concentrated on creating standardised protocols to ensure patients receive the same opportunity for education and improved outcomes (Roeung et al., 2024; van Loon et al., 2020). Whilst such protocols provide consistency, they can overlook the diverse needs of individual patients. As healthcare services have moved towards a ‘person-centred model of care’ (Byrne, 2025), it is essential to shift our focus toward strategies that are holistic, compassionate, responsive to patients’ unique circumstances and offer shared decision making to deliver positive outcomes.

The International Ostomy Association’s Charter of Ostomates Rights (2007) outlines the rights of patients with an ostomy to preoperative counselling, appropriate stoma siting, and access to experienced (ostomy) nursing care preoperatively and postoperatively. Whilst many hospitals offer these services, the challenge for nurses and other clinicians is how to deliver effective and personalised education with increasingly shortened hospital stays and limited resources. This has prompted interest in alternative educational methods, including digital tools such as telehealth (Özkaya and Harputlu, 2024), mobile applications (commonly known as ‘apps’) (Ketelaers et al., 2023), and social media (Linz et al., 2025).

Previous literature reviews have examined educational strategies for patients with intestinal ostomies, often focusing on specific aspects such as preoperative education (Shi et al., 2025), multimedia-based interventions (Qiao et al., 2024), randomised controlled trials (Faury et al., 2017), selected outcomes (Phatak et al., 2014), quantitative studies (Danielsen et al., 2013) or literature published prior to 2020 (Chapman et al., 2020). Whilst these reviews provide valuable insights, their targeted focus means that evidence related to the broader range of educational strategies used across the perioperative pathway has not been recently synthesised within a single review. Given the diversity of educational strategies and heterogeneity of outcomes reported across studies (Danielsen et al., 2013; Faury et al., 2017; Qiao et al., 2024), a comprehensive summary is needed to clarify existing evidence and identify gaps in research. Therefore, this scoping review was undertaken to identify the global educational strategies for patients undergoing colorectal ostomy surgery and to summarise the outcomes measured in these studies.

## 2 Method

### 2.1 Study design

Scoping reviews are used to explore, map, and summarise the evidence, and inform future research (Tricco et al., 2016). A scoping review was considered an appropriate method for exploring the topic as it can examine a broad or complex topic area to identify gaps in knowledge, key concepts, sources and gaps in the literature (Khalil et al., 2025). The five-stage methodological framework of Arksey and O'Malley (2005) with modifications by Levac et al. (2010) guided the conduct of the review. The PRISMA-ScR (Preferred Reporting Items for Systematic Reviews and Meta-Analyses extension for Scoping Reviews) checklist (Tricco et al., 2018) served as the reporting guideline for this review.

### 2.2 Research questions

The following research questions guided this review:

(1) What forms of education are offered to patients undergoing colorectal ostomy surgery?
(2) What are the outcomes of the education?

### 2.3 Eligibility criteria

The Participants, Concept, and Context (PCC) framework (Peters et al., 2020) was adopted to determine studies eligible for inclusion in the review. The PPC framework is recommended as a guide to building clear eligibility criteria and identify any gaps or missed criteria (Pollock et al., 2023).

#### 2.3.1 Participant

The review consists of studies that included nurses and other health care professionals who had been involved in the counselling/education of adults undergoing surgery for the creation of an ileostomy or colostomy. Furthermore, studies that included the family members or the caregivers of the patients were also included in the review due to their frequent involvement in ostomy care. Studies involving the education of paediatric patients were excluded due to the difference in educational content between adults and children.

#### 2.3.2 Concept

The concept of interest was the strategies identified in educational interventions for colorectal ostomy surgery in the perioperative context.

#### 2.3.3 Context

For this review, the term ‘preoperative’ is the time from when the patient is booked for the operation until the time surgery commences, ‘postoperative’ is the time from when surgery concludes until the discharge of the patient from hospital to community, and ‘perioperative’ is the period of time surrounding surgery, encompassing both preoperative and postoperative periods, from the time surgery is booked until up to six months post-surgery.

### 2.4 Search strategy

Four databases were searched: MEDLINE (via OVID), CINAHL (via OVID), PsycINFO, and Scopus. Key words were developed using MeSH Headings, tailored to each database; search strings were developed with Boolean operators, truncations and subject headings. University and health service librarians supported the authors in the development of the search strategy for each database. Refer to Appendix I for the Search Strategy used for each database. The reference lists of included articles were reviewed to identify additional studies.

Peer-reviewed research articles published up until 19 November 2024 were included with no further restrictions on year of publication or language or study design. Grey literature, quality improvement projects, reviews and letters to the editor were excluded from the search.

### 2.5 Data extraction

**Fig. 1:**
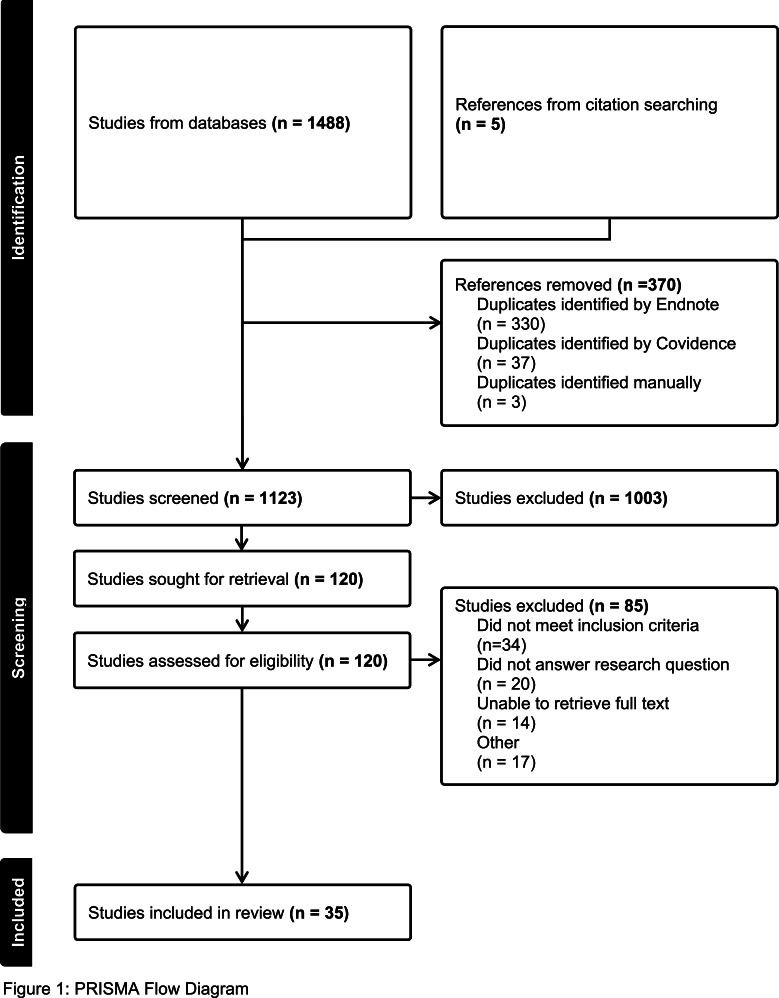
PRISMA flow diagram.

A total of 1488 identified studies were uploaded into EndNote v20 (The Endnote Team, 2013) and Covidence (2025); duplicates were removed. Two members of the research team (MB, KG) independently screened the titles and abstracts in Covidence, then retrieved the full texts to ensure articles met the selection criteria. Articles were excluded if the full text was unable to be retrieved. A third team member (WS) was available to resolve any conflict in decisions, resulting in 35 studies included in the final review. A PRISMA flow diagram (Page et al., 2021) was used to record the retrieval and selection process (See Fig. 1).

### 2.6 Data charting

Preliminary data charting was undertaken, by the first author, in Covidence, then exported to Excel where it was refined and expanded, with input from all authors. The chart of included studies was developed with details of authors' names, publication year, country, study design, aim, sample, education, phase, setting, and key findings (see Supplementary Table 1). Grimes and Schulz’s (2002) criteria were used to classify the study design as either randomised controlled trial (RCT), non-RCT, analytical study or descriptive study. The data chart was reviewed and agreed upon by all team members. No specific critical appraisal scoring tool was used when reading the included studies.

## 3 Results

Thirty-five articles, from 18 countries, published between 2004 and 2024 were included (see Supplementary Table 1. Characteristics of included studies). Eighteen were RCTs; sample sizes ranged from 12 to 4201 ostomates with colostomy or ileostomy. Nurses were involved in education delivery in 30 studies, alongside other professionals such as surgeons, dieticians, psychologists, and sexologists. Five studies did not disclose the type of education provider (Abdelmohsen, 2020; Alenezi and Mansour, 2016; Çakır and Özbayır, 2018; Lo et al., 2010, 2011). Three studies included peer educators with lived experience of an ostomy (He et al., 2021; Song et al., 2021; Wang et al., 2024).

### 3.1 Research question 1. Educational strategies

Education was delivered face-to-face in 32 studies and online in the remaining three (Ketelaers et al., 2023; Ko et al., 2023; Zhou et al., 2023). Face-to-face education was provided individually to patients, with five studies additionally offering group sessions (Abdelmohsen, 2020; Danielsen and Rosenberg, 2014a; He et al., 2021; Stokes et al., 2017; Yeo and Park, 2023). Seventeen studies mentioned patients received written materials (booklets, pamphlets) as part of their education program. Multimedia tools (videos, apps) were used in 19 studies; four of those studies involved the use of an interactive app to provide patient education (Ketelaers et al., 2023; Song et al., 2021; Yiğitoğlu and Şendir, 2021; Zhou et al., 2023).Twelve studies included preoperative practical education of the ostomy appliance as well as theoretical education with four studies giving participants the opportunity to take the products home to practice with (Forsmo et al., 2016; Gibbins, 2011; Hughes et al., 2020; Younis et al., 2012). Two studies additionally provided participants with mock stomas on which to practice for a life-like experience (Hughes et al., 2020; Younis et al., 2012).

Personalised education was evident in eight studies (Çakır and Özbayır, 2018; Duque et al., 2023; Gonella et al., 2019; Ketelaers et al., 2023; Kittinouvarat et al., 2011; Lin et al., 2024; Özkaya and Harputlu, 2024; Wang et al., 2024). These studies showed flexibility in their delivery of education and communication with the patient, by personalising education plans to account for age, literacy level or social environment (Gonella et al., 2019; Kittinouvarat et al., 2011), tailoring support with additional phone calls (Özkaya and Harputlu, 2024), selecting stoma sites based on patients’ individual characteristics and lifestyle (Çakır and Özbayır, 2018), and changing content based on the needs of patients in the group (Duque et al., 2023; Wang et al., 2024).

Education was provided at different stages of the patients’ surgical pathway. Eighteen studies included education preoperatiely, while most studies (*n* = 30) described postoperative education, with 13 incorporating both phases (Refer to Supplementary Table 1). Preoperatively, common topics included stoma siting (Çakır and Özbayır, 2018; Danielsen and Rosenberg, 2014a; Koc et al., 2023; Lim et al., 2019; Yeo and Park, 2023), gastrointestinal anatomy (Heidari-Beni et al., 2022; Lin et al., 2024; Lo et al., 2010; Yiğitoğlu and Şendir, 2021), and explanation of the proposed surgery (Forsmo et al., 2016; Kittinouvarat et al., 2011; Lo et al., 2010). With planned surgery, some studies offered patients ostomy products with which to practice (see preceding paragraph). Postoperatively, education focused on developing patient competence, confidence and/or independence in ostomy care (Abdelmohsen, 2020; Chaudhri et al., 2005; Danielsen and Rosenberg, 2014a; Duque et al., 2023; Forsmo et al., 2016; García-Cabrera et al., 2023; Gibbins, 2011; Gonella et al., 2019; He et al., 2021; Heidari-Beni et al., 2022; Ketelaers et al., 2023; Kittinouvarat et al., 2011; Ko et al., 2023; Koc et al., 2023; Lim et al., 2019; Lin et al., 2024; Özkaya and Harputlu, 2024; Seo, 2018; Song et al., 2021; Stokes et al., 2017; van Pelt et al., 2024; Wang et al., 2021; Yeo and Park, 2023; Yiğitoğlu and Şendir, 2021). The prevention and management of complications, such as peristomal skin irritation and dehydration were frequently addressed (Abdelmohsen, 2020; Crawford et al., 2012; Gonella et al., 2019; Ketelaers et al., 2023; Seo, 2018; Wang et al., 2021; Yeo and Park, 2023; Yiğitoğlu and Şendir, 2021). Following discharge, education focused on reinforcing self-care skills and independence (Kittinouvarat et al., 2011; van Pelt et al., 2024; Zhou et al., 2023), and supporting adjustment to life with an ostomy (Danielsen and Rosenberg, 2014a; Duque et al., 2023; Özkaya and Harputlu, 2024).

Several studies incorporated family or caregiver involvement in patient education across the surgical pathway. Family members were invited to attend hospital-based education in 5 studies (Gonella et al., 2019; He et al., 2021; Lin et al., 2024; Lo et al., 2010; Stokes et al., 2017), with timing varying across studies: preoperative (Stokes et al., 2017), both preoperative and postoperative (Gonella et al., 2019), and postoperative only (He et al., 2021; Lin et al., 2024; Lo et al., 2010). Three studies (Heidari-Beni et al., 2022; Ko et al., 2023; Lo et al., 2011) provided educational resources for family members to view at home postoperatively, however, did not explicitly invite them to the in-hospital education sessions. Only one study (He et al., 2021) evaluated the impact of family attendance at an educational session post-discharge, reporting reduced peristomal dermatitis, while Ko et al. (2023) was the only study to recommend further research on the impact of caregiver involvement on patient outcomes.

Eight studies reported patient involvement in intervention development, with varying levels of engagement. In six studies (Çakır and Özbayır, 2018; Heidari-Beni et al., 2022; Ketelaers et al., 2023; Ko et al., 2023; Lin et al., 2024; Lo et al., 2010; Yiğitoğlu and Şendir, 2021), involvement was primarily consultative, with patients contributing to content review, usability testing, and refinement of educational materials. One study (Lo et al., 2010) limited participation to pilot testing of outcome measures and face validity of questions. Only two studies (Song et al., 2021; Wang et al., 2024) demonstrated co-design approaches. These reported statistically significant improvements in self-efficacy, self-management ability, and reduced complication rates (Song et al., 2021), as well as improvements in quality of life, self-care ability, psychological adaptation, and reduced complication rates (Wang et al., 2024).

Six studies incorporated peer-led or peer-supported components within intervention delivery, including the use of peer educators, lay teachers, or experienced ostomy patients providing structured support or sharing lived experience (Danielsen and Rosenberg, 2014a; Gibbins, 2011; He et al., 2021; Lin et al., 2024; Song et al., 2021; Wang et al., 2024). Interventions including peer support reported statistically significant improvements in self-care proficiency and quality of life (Ko et al., 2023; Wang et al., 2024), reduced complication rates (He et al., 2021; Wang et al., 2024), and improvements in self-efficacy and self-management ability (Song et al., 2021).

### 3.2 Research question 2. Outcomes measured

Outcomes measured were categorised under: knowledge and skills; quality-of-life and psychosocial wellbeing; clinical outcomes; patient satisfaction; and healthcare resources and costs (See Table 1).

**Table 1:** Outcomes measured.

Outcomes measured		Reference (first author)
Knowledge and skills	Self-care knowledge	Abdelmohsen 2020; Alenezi 2016; Çakır 2018; Kittinouvarat 2011; Lo 2010; Lo 2011; Seo 2018; Wang 2021; Wang 2024; Yeo 2023;
	Self-care skills	Abdelmohsen 2020; Chaudri 2005; Crawford 2012; Gibbins 2011; Kittinouvarat 2011;Ko 2023; Koc 2023; Lim 2019; Seo 2018; van Pelt 2024; Wang 2021; Wang 2024; Yeo 2023; Zhou 2023
Quality of life and psychosocial Skills	Quality of life	Danielsen and Rosenberg, 2014a; Duque 2023; Forsmo 2016;Ko 2023; Koc 2023; Lim 2019; Lin 2024; Wang 2024;
	Anxiety and depression	Çakır 2018; Chaudri 2005; Koc 2023; Lim 2019; Yeo 2023
	Stoma self-efficacy	Lim 2019; Lin 2024; Lo 2010; Lo 2011; Ozkaya 2024; Seo 2018; Song 2021; Yiğitoğlu 2021
	Ostomy adjustment and acceptance	Danielsen and Rosenberg, 2014a; Heidari-Beni 2022; Lim 2019; Ozkaya 2024; Wang 2024; Yiğitoğlu 2021; Zhou 2023
Clinical outcomes	Postoperative complications	Alenezi 2016; Forsmo 2016; García-Cabrera 2023; He 2021; Lin 2024; Song 2021; Stokes 2017; Tweed 2021; Wang 2024; Yeo 2023; Yiğitoğlu 2021
	Unplanned hospital readmissions	Forsmo 2016; García-Cabrera 2023; Gonella 2019; Hughes 2020; Iqbal 2017; Lin 2024; Stokes 2017; Tweed 2021
	Length of stay (LOS)	Chaudhri 2005; Forsmo 2016; García-Cabrera, 2023; Hughes 2020; Iqbal 2017; Lim 2019; Lin 2024; Stokes 2017; Tweed 2021; Yeo 2023; Younis 2012
Patient satisfaction	Satisfaction with intervention	Gibbins 2011; Iqbal 2017; Kittinouvarat 2011; Lin 2024; Song 2021; Wang 2024; Yiğitoğlu 2021
Healthcare resources and costs	Cost of care	Chaudri 2005; Iqbal 2017; Lo 2010
	Nursing workload	Chaudri 2005; Ketelaers 2023; van Pelt 2024

#### 3.2.1 Knowledge and skills

Ostomy self-care knowledge was measured in 10 studies (Abdelmohsen, 2020; Alenezi and Mansour, 2016; Çakır and Özbayır, 2018; Kittinouvarat et al., 2011; Lo et al., 2010, 2011; Seo, 2018; Wang et al., 2021, 2024; Yeo and Park, 2023), all showing improvement regardless of timing or modality of the education. Ostomy self-care skills were evaluated in 14 studies with 11 studies (Abdelmohsen, 2020; Chaudhri et al., 2005; Crawford et al., 2012; Ko et al., 2023; Koc et al., 2023; Seo, 2018; van Pelt et al., 2024; Wang et al., 2021, 2024; Yeo and Park, 2023; Zhou et al., 2023) finding that a patient's ability to perform their own stoma care proficiently was attributed to education exposure. Three studies (Gibbins, 2011; Kittinouvarat et al., 2011; Lim et al., 2019) did not show significant improvement in self-care skills. Although overall outcomes were not significantly improved, Kittinouvarat et al. (2011) observed that patients who received preoperative hands-on ostomy training and an educational DVD required less postoperative instruction. Similar benefits were reported by Koc et al. (2023) and Chaudhri et al. (2005) in their RCTs, who reported significantly greater independence in ostomy care with the addition of preoperative education to postoperative education. The opportunity to handle the ostomy appliance before surgery enabled faster achievement of care proficiency, which in turn shortened hospital stays (Chaudhri et al., 2005; Koc et al., 2023). Despite significantly higher rates of ostomy acceptance in Lim et al.’s (2019) RCT, the introduction of a preoperative education session and addition of five follow-up phone calls did not improve self-care skills.

#### 3.2.2 Quality of life and psychosocial skills

Quality-of-Life (QoL) was measured in eight studies, with six (Danielsen and Rosenberg, 2014a; Duque et al., 2023; Ko et al., 2023; Koc et al., 2023; Lin et al., 2024; Wang et al., 2024) showing an improvement in QoL after education interventions. All eight studies included postoperative education. Anxiety and depression were measured in five studies, with three (Çakır and Özbayır, 2018; Koc et al., 2023; Yeo and Park, 2023) showing statistically significant reductions following additional preoperative education. When comparing the anxiety scores following preoperative education to scores following postoperative education, Yeo and Park (2023) found preoperative education to be more effective in lowering patient anxiety.

Stoma Self-Efficacy, described as the belief in one’s own abilities to manage their ostomy and the associated challenges (Aker and Karazeybek, 2025, p.2), was evaluated in eight studies. All of these studies included postoperative education, with seven (Lin et al., 2024; Lo et al., 2010, 2011; Özkaya and Harputlu, 2024; Seo, 2018; Song et al., 2021; Yiğitoğlu and Şendir, 2021) reporting statistically significant improvements. Adjustment and acceptance were measured in seven studies, all reporting significant improvements, particularly in the interventions that used multimedia (Heidari-Beni et al., 2022; Yiğitoğlu and Şendir, 2021; Zhou et al., 2023) and peer-led education (Wang et al., 2024). Long-term benefits (psychosocial adjustment and self-efficacy) up to six months postoperatively were noted with ongoing app-based education (Yiğitoğlu and Şendir, 2021).

#### 3.2.3 Clinical outcomes

Postoperative complications were monitored in 11 studies. These included major and minor complications, and ostomy-related complications such as leakage, peristomal skin irritation, stoma retraction, and parastomal hernia. Eight studies (Alenezi and Mansour, 2016; He et al., 2021; Lin et al., 2024; Song et al., 2021; Stokes et al., 2017; Tweed et al., 2021; Wang et al., 2024; Yeo and Park, 2023) showed a decrease in peristomal complications with only postoperative education (*n* = 5); both preoperative and postoperative education (*n* = 2); and only preoperative education (*n* = 1). Peristomal skin irritation (peristomal dermatitis or hyperemic lesion) remained a common minor complication for ileostomy and colostomy patients (Alenezi and Mansour, 2016; Forsmo et al., 2016; He et al., 2021; Stokes et al., 2017; Yeo and Park, 2023). The most common major complication was dehydration associated with high-output ileostomies which often resulted in readmission (Forsmo et al., 2016). For these reasons, skin care and hydration were regularly included as topics of education.

Unplanned hospital readmission rates were monitored in eight studies; three finding the inclusion of postoperative education and follow-up phone calls post-discharge was associated with fewer unplanned readmissions (Gonella et al., 2019; Iqbal et al., 2017; Lin et al., 2024). Length of stay (LOS), measured in 11 studies, was considered ‘the number of days from surgery to hospital discharge’. Of the 11 studies, seven reported a reduction in LOS (Chaudhri et al., 2005; Forsmo et al., 2016; Hughes et al., 2020; Iqbal et al., 2017; Tweed et al., 2021; Yeo and Park, 2023; Younis et al., 2012). Six studies (Chaudhri et al., 2005; Forsmo et al., 2016; Hughes et al., 2020; Tweed et al., 2021; Yeo and Park, 2023; Younis et al., 2012) reported preoperative education allowed the patients to be more responsive to knowledge and skill development after surgery resulting in shorter LOS. Three studies (Forsmo et al., 2016; Hughes et al., 2020; Tweed et al., 2021), offered preoperative and postoperative education within an Enhanced Recovery After Surgery (ERAS) program; all resulted in reduced LOS.

#### 3.2.4 Patient satisfaction

Satisfaction with intervention and support was measured in seven studies. All but one (Gibbins, 2011) reported high or excellent satisfaction regardless of the type or timing of the education. Of the five studies that measured satisfaction after a multimedia intervention (Gibbins, 2011; Lin et al., 2024; Song et al., 2021; Wang et al., 2024; Yiğitoğlu and Şendir, 2021), one study Gibbins (2011), reported patient dissatisfaction due to poor comprehension of multimedia content, preferring the postoperative personal instruction to the preoperative DVD. The other four multimedia-based interventions consistently reported high or improved satisfaction compared with standard care. Satisfaction in these multimedia studies was largely attributed to the clarity, vividness, and accessibility of digital formats such as videos, animations, mobile apps and visual education platforms, with participants describing the content as intuitive and easy to revisit. The two non-multimedia studies (Iqbal et al., 2017; Kittinouvarat et al., 2011), which relied on counselling and structured follow-up, also achieved excellent satisfaction, with participants valuing the personalised support and frequent human contact.

#### 3.2.5 Healthcare resources and costs

Cost of care was evaluated in three studies, all reporting that educational interventions were cost-efficient due to: a reduction in LOS (Chaudhri et al., 2005); fewer readmissions (Iqbal et al., 2017); and an improvement in patient and family knowledge and skills (Lo et al., 2010). Both preoperative and postoperative strategies led to cost savings for the health service and the patient. For example, Chaudhri et al. (2005) reported savings of UK£1119 per patient with preoperative home visits and Iqbal et al. (2017) showed a reduction in readmission costs by US$63,821 per patient with daily postoperative phone calls from a clinician.

Three studies examined nursing workload. Ketelaers et al. (2023) and van Pelt et al. (2024) reported fewer post-discharge nurse contacts or visits when preoperative education sessions were combined with mobile app use, especially among younger patients (Ketelaers et al., 2023), or after practice with the ostomy appliance (van Pelt et al., 2024). Chaudhri et al. (2005) also found that preoperative community education reduced the workload on ostomy support services during the first six weeks following discharge compared to patients who received postoperative education only.

## 4 Discussion

This scoping review identified 35 studies across 18 countries, underscoring the global relevance of education in colorectal ostomy surgery. Compared with previous syntheses (Chapman et al., 2020; Colwell and Gray, 2007; Danielsen et al., 2013; Faury et al., 2017; Qiao et al., 2024; Shi et al., 2025), this review captures more recent developments, reflecting a growing recognition of the impact of ostomy education on patient outcomes and health system performance. Consistent with earlier reviews, the findings support the role of education in improving: patient psychosocial skills (Danielsen et al., 2013; Faury et al., 2017); complications (Qiao et al., 2024; Shi et al., 2025); cost efficiencies (Danielsen et al., 2013); and length of stay (Chapman et al., 2020). This review extends previous work (Chapman et al., 2020; Danielsen et al., 2013; Faury et al., 2017; Phatak et al., 2014; Qiao et al., 2024; Shi et al., 2025) by identifying a shift toward multi-modal and technology-enabled delivery, alongside growing emphasis on personalised and participatory approaches, including co-design and peer-supported education. Education needs to focus on patients’ self-care, confidence and competence. To this end, a recent qualitative study explored nurses’ perspectives as to how education could be designed, resourced and delivered to assist patients to adapt to living with a stoma (Soares-Pinto, 2025). This discussion also highlights nurse-led education, caregiver involvement and delivery across the perioperative continuum.

Consistent with earlier reviews (Faury et al., 2017; Phatak et al., 2014; Shi et al., 2025) face-to-face teaching remained the most common method of ostomy education. However, this review identified a shift toward multi-modal approaches, combining traditional in-person education with digital and remote delivery methods to meet the demands of shorter hospital stays and increasingly diverse patient populations. Evidence suggests that integrating multiple educational strategies enhances patient preparedness and supports a broader range of outcomes, including knowledge acquisition, self-care ability, psychological adjustment, complication reduction, readmissions, and length of stay. These findings align with broader evidence indicating that multi-modal interventions, incorporating formats such as mobile apps, messaging platforms, and web-based resources alongside face-to-face teaching, can optimise engagement and learning across diverse patient groups (Qiao et al., 2024; Yang and Van Stee, 2019).

There was increasing emphasis on personalised and flexible education tailored to individual needs, including health literacy, self-care capacity, and social context. While structured programmes provide consistency, flexible delivery appears critical to effective learning.

Eight studies included in this review did in fact highlight the growing emphasis on tailoring education to individual patient needs. Recent literature (Ricci et al., 2022) also acknowledges this transition to personalised education, recognising both the importance of patient-centred approaches and the limitations of overly rigid, check-list driven frameworks (Gibbins, 2011). However, the issue may not lie in standardisation itself, but rather in how it is implemented. Structured programmes can provide a consistent framework of essential educational content, timing, and clearly defined professional roles for ostomy nurses. Within this framework, however, the reviewed literature suggests that educational delivery may be most effective when it remains flexible and responsive to each patient’s literacy, autonomy, social context, and evolving needs.

Within this broader shift, technology-enabled education emerged as a key component. Digital tools were associated with improved knowledge and skills (Abdelmohsen, 2020; Lo et al., 2010, 2011; Wang et al., 2021, 2024; Yeo and Park, 2023), increased adjustment to ostomy (Heidari-Beni et al., 2022; Özkaya and Harputlu, 2024; Yiğitoğlu and Şendir, 2021), reduced complications (Chaudhri et al., 2005; Lin et al., 2024; Song et al., 2021; Wang et al., 2024; Yeo and Park, 2023), fewer readmissions (Lin et al., 2024), and shorter length of stay (Chaudhri et al., 2005; Yeo and Park, 2023; Younis et al., 2012). Mobile apps, in particular, supported ongoing education for up to six months postoperatively (Ketelaers et al., 2023; Song et al., 2021; Yiğitoğlu and Şendir, 2021; Zhou et al., 2023), with large-scale implementation demonstrated by Zhou et al. (2023). Technology also enabled education to extend beyond acute care settings, with apps such as StoManager supporting preoperative and postoperative learning (Ketelaers et al., 2023). Emerging approaches, including virtual reality (Shepherd et al., 2024) and social media-based education (Azer et al., 2022; Linz et al., 2025), further highlight the expanding role of digital platforms in patient education. Despite these advantages, limitations relating to digital access and literacy were evident. Several studies restricted participation to individuals with smartphones, internet access, or adequate literacy, limiting generalisability (Ketelaers et al., 2023; Yiğitoğlu and Şendir, 2021; Zhou et al., 2023). Exclusion of individuals with communication impairments or limited digital capability highlights the risk of inequitable access. Digital resources, although effective at improving patient outcomes and reducing post-discharge service utilisation, are costly to develop and require regular use and updating to ensure sustainability (An et al., 2023).

In parallel to these digital developments, the adoption of co-designed and peer-led models of education in colorectal ostomy patients were evident. The findings suggest that participatory approaches, particularly co-design, may extend the impact of educational interventions beyond knowledge acquisition to influence psychosocial outcomes. While expert-led interventions improved procedural knowledge and clinical indicators, their effects on psychological adaptation and long-term stoma acceptance were less consistent. In contrast, co-designed interventions (Song et al., 2021; Wang et al., 2024) demonstrated improvements across behavioural and psychosocial domains, including self-efficacy and adaptation. This is consistent with broader evidence indicating that co-design enhances relevance, acceptability, and alignment with patient needs by integrating lived experience into intervention development (Morley et al., 2024). Participatory approaches may also support empowerment and self-efficacy by positioning patients as active contributors (Harrison et al., 2019).However, the small number of co-designed studies limits generalisability and highlights the need for further evaluation.

Peer-led and peer-supported components during intervention also emerged as a key feature of education delivery. Several studies (Danielsen and Rosenberg, 2014a; Gibbins, 2011; He et al., 2021; Lin et al., 2024; Song et al., 2021; Wang et al., 2024) included peer educators or experienced patients who contributed to discussions and skill development through sharing their lived experience. These studies showed improvements in self-management, quality of life, and complication rates, suggesting that lived experience may enhance engagement and address practical (self-efficacy) and social (psychosocial adjustment) aspects of living with an ostomy. This contrasts with earlier literature reporting limited use of peer-led models (Danielsen et al., 2013) and aligns with increasing emphasis on person-centred care and the integration of experiential knowledge in healthcare delivery (Australian Commission on Safety and Quality in Health Care, 2011; World Health Organization, 2025).

Consistent with prior reviews, nurses, often described as ostomy or stoma specialists, remained the primary educators and were associated with improved clinical and economic outcomes. Their involvement was associated with higher patient satisfaction (Lin et al., 2024; Wang et al., 2024; Yiğitoğlu and Şendir, 2021), fewer peristomal skin complications (Alenezi and Mansour, 2016; Stokes et al., 2017), reduced readmissions due to dehydration (Gonella et al., 2019), and lower healthcare costs (van Pelt et al., 2024). Nurses’ contribution to integrating digital tools and coordinating ongoing support in ostomy education is clearly reflected in the number of studies designed and delivered by nurses. Although earlier studies rarely addressed the impact of technology on the workload of the ostomy specialist nurse, this review found that mobile apps and multimedia tools may reduce post-discharge nurse contacts and the need for community services (Ketelaers et al., 2023). These findings reinforce the ongoing importance of nurse-led education and ostomy-trained nurses in enhancing care quality while reducing avoidable healthcare costs (Danielsen and Rosenberg, 2014b). They also align with international evidence that nurses working to their full scope and in advanced practice roles achieve improved clinical outcomes, fewer complications, and high patient satisfaction (Kilpatrick et al., 2024). The findings suggest high patient satisfaction can be achieved through both multimedia-enhanced education and intensive interpersonal support. Multimedia tools appear to enhance engagement and accessibility, while non-multimedia approaches were valued for interpersonal connection and tailored guidance. Future interventions may benefit from integrating both elements to personalise patient experience and accommodate diverse learning preferences.

The reviewed literature indicates that family and caregiver involvement may play an important role in patient education across the surgical pathway. In this review, interventions incorporating patient and family input and peer educators, reported significantly higher satisfaction (Ketelaers et al., 2023; Wang et al., 2024), greater self-care proficiency (Ko et al., 2023; Wang et al., 2024), improved quality of life (Ko et al., 2023; Wang et al., 2024) and reduced complications (He et al., 2021; Wang et al., 2024). Previous research has recognised that family involvement can reinforce self-care learning during the transition from hospital to home, support emotional adjustment, and facilitate shared care when patient autonomy is limited or evolving (Chartrand et al., 2023). Conversely, the absence of a caregiver has been identified as a significant risk factor for hospital readmission among ileostomy patients (Fish et al., 2017). Despite these recognised benefits, caregiver involvement in patient education remains underrepresented in the literature.

The timing of the education featured prominently across the reviewed studies, indicating benefits at multiple stages of the surgical pathway. While education at any stage was associated with improved knowledge and self-care, preoperative education was particularly linked to reductions in anxiety and depression (Çakır and Özbayır, 2018; Koc et al., 2023; Yeo and Park, 2023). Postoperative education was similarly noted in the literature for supporting long-term adjustment and providing real-time support (Qiao et al., 2024, p.11), which was reflected in several included studies (Heidari-Beni et al., 2022; Özkaya and Harputlu, 2024). Studies combining both preoperative and postoperative education reported a broader range of outcomes, including accelerated skill acquisition and reduced LOS (Chaudhri et al., 2005; Tweed et al., 2021; Yeo and Park, 2023; Younis et al., 2012).

Finally, the findings of this review support education led by nurses as a continuous process spanning preoperative preparation, postoperative learning, and post-discharge consolidation of self-care. This continuity is particularly relevant within Enhanced Recovery After Surgery (ERAS) pathways, where shorter hospital stays may limit opportunities for reinforcement prior to discharge. For example, in an Australian retrospective study by Tan et al. (2025), the absence of ostomy education on weekends was the primary cause of delayed discharge for patients, adding financial burden to the health service. In this context, ongoing post-discharge education and support by highly skilled nurses are highlighted in the literature (Wainwright et al., 2022), with long term education associated with improved psychological adjustment and resilience, as well as reduced reliance on community services (Chaudhri et al., 2005; Ketelaers et al., 2023; van Pelt et al., 2024). Collectively, these studies demonstrate the value of ostomy education spanning the entire surgical journey, supporting both patient care and health service efficiency.

### 4.1 Limitations and strengths

Limitations of the included studies mirror those noted in previous reviews, including small sample sizes, single-centre designs, and short follow-up periods, which limit generalisability and the assessment of long-term outcomes. Only one of the 35 studies was multi-centre, and few evaluated long-term outcomes or patient experiences, a gap also highlighted in earlier reviews. Considerable heterogeneity in intervention components, timing, and outcome measures limits comparison between studies and precludes meta-analysis, as noted in previous syntheses (Chapman et al., 2020; Danielsen et al., 2013; Faury et al., 2017; Qiao et al., 2024; Shi et al., 2025). Many interventions focused solely on preoperative or postoperative education, despite evidence that combining both offers broader benefits.

A strength of this review was the transparent documentation of the review process and the compliance with rigorous methodological and reporting frameworks by Arksey and O'Malley (2005) and PRISMA-ScR guidelines (Tricco et al., 2018). Additionally, the identification of numerous RCTs provided high level evidence compared to previous reviews with fewer trials.

### 4.2 Implications for future research

While scoping reviews do not provide recommendations for practice or policy, they can identify knowledge gaps and describe implications for future research (Pollock et al., 2021).

This scoping review highlights several important gaps in the evidence base for stoma education. Although technology-enabled education is widely reported, evidence regarding its long-term effectiveness, sustainability, and equity of access is limited. Future studies should include populations with varying levels of digital literacy and access to improve their generalisability. The gap in qualitative research exploring stoma patients’ perspectives on perioperative education, particularly across the transition from hospital to community care was evident. Greater attention to patient, carer, and peer experiences may support the development of educational programs that are more responsive to patient needs.

Participatory approaches, including co-design and peer-supported interventions, were evident but inconsistently defined. There is limited understanding of how these approaches influence behavioural and psychosocial outcomes of ostomy patients and their carers, and greater consistency in reporting is needed. Growing evidence of co-designed education suggests this is a growing research priority rather than a longstanding gap. Comparative studies examining different models of peer involvement, including intensity and mode of delivery, would provide valuable insight. Future research should prioritise rigorous evaluation of the usability, effectiveness and cost-effectiveness of scalable, nurse-led educational interventions to inform resource allocation and support sustainable and contemporary models of care.

Education that is flexible, individualised, and delivered across the perioperative pathway appears most consistent with improved patient outcomes, however, limited evidence exists on the continuity of education and the role of family and caregiver involvement in supporting long-term patient outcomes. The exclusion of emergency surgery patients in several studies further indicates a need to explore flexible education that accommodates limited preoperative time and greater postoperative and post-discharge support. Finally, there was limited evidence of objective measurements of patients’ competence with caring for their stoma. Future research could prioritise exploring the impact of education on patients’ actual behaviour.

## 5 Conclusion

The reviewed studies collectively emphasise the critical role of comprehensive and tailored education and support in improving the lives of people living with an ileostomy or colostomy. Effective preoperative education can alleviate anxiety, reduce length-of-stay, and better prepare patients for surgery and life with a stoma. Postoperative education and ongoing support are essential for enhancing self-care skills, promoting psychological adjustment, improving quality of life, and preventing and managing complications. The increasing use of multi-modal and technology-enabled approaches, alongside practical tools and peer-supported learning, reflects a shift toward more accessible and patient-centred models of education. These approaches, often delivered by ostomy specialist nurses, contribute to improved patient experiences and more efficient use of health services. Overall, the findings support a model of ostomy education that is continuous, personalised, and delivered across the perioperative pathway, integrating multiple delivery methods to improve patient and health service outcomes.

## Disclosures and acknowledgements

### Ethical Considerations

This scoping review involves analysis of existing literature and does not involve direct data collection from human subjects. Therefore, ethical approval was not required.

## Funding

This project was undertaken as part of a study with $66,193 funding from the Townsville Hospital and Health Service Study Education and Research Trust Account (SERTA) Fund, Grant THHSSERTA_RSFG3_2024. The funder was not involved in any part of the study (including data analysis) or writing of the manuscript.

## CRediT authorship contribution statement

**Marina Bogiatzis:** Writing – review & editing, Writing – original draft, Visualization, Project administration, Investigation, Funding acquisition, Formal analysis, Conceptualization. **Karen Gerrard:** Writing – review & editing, Writing – original draft, Investigation, Formal analysis. **Wendy Smyth:** Writing – review & editing, Supervision, Project administration, Formal analysis.

## Declaration of competing interest

The authors declare that they have no conflicting interests.

## Appendix Supplementary materials

Image, application 1

Image, application 2

Image, application 3

## References

[bib0001] Abdelmohsen S. (2020). Effectiveness of structured education on patient's knowledge and practice regarding colostomy care. Asia Pac. J. Oncol. Nurs..

[bib0002] Aker F.Z., Karazeybek E. (2025). Relationship between perceived social support and stoma self-efficacy in permanent colostomy patients: a correlational study. J. Eval. Clin. Pract..

[bib0003] Alenezi A., McGrath I., Kimpton A., Livesay K. (2021). Quality of life among ostomy patients: a narrative literature review. J. Clin. Nurs..

[bib0004] Alenezi A.N., Mansour E.A. (2016). Impact of stoma care education in minimizing the incidence of stoma skin complications. Bahrain Med. Bull..

[bib0005] An Q., Kelley M.M., Hanners A., Yen P.-Y. (2023). Sustainable development for mobile health apps using the human-centered design process. JMIR Form. Res..

[bib0006] Arksey H., O'Malley L. (2005). Scoping studies: towards a methodological framework. Int. J. Soc. Res. Methodol..

[bib0007] Australian Commission on Safety and Quality in Health Care (2011). https://www.safetyandquality.gov.au/sites/default/files/migrated/PCC_Paper_August.pdf.

[bib0008] Ayaz-Alkaya S. (2019). Overview of psychosocial problems in individuals with stoma: a review of literature. Int. Wound J..

[bib0009] Azer S.A., AlKhawajah N.M., Alshamlan Y.A. (2022). Critical evaluation of YouTube videos on colostomy and ileostomy: can these videos be used as learning resources?. Patient Educ. Couns..

[bib0010] Byrne A.-L. (2025). Two sides of the same coin: person-centred systems versus person-centred nursing practice. Theory, barriers and opportunities. J. Res. Nurs..

[bib0011] Çakır S.K., Özbayır T. (2018). Assessment of patient anxiety levels before and after stoma surgery. Turk. J. Colorectal Dis..

[bib0012] Chapman S.J., Helliwell J.A., Lonsdale M.D.S., Tiernan J.P., Jayne D.G. (2020). Patient education about recovery after colorectal surgery: systematic scoping review. Colorectal Dis..

[bib0013] Chartrand J., Shea B., Hutton B., Dingwall O., Kakkar A., Chartrand M., Poulin A., Backman C. (2023). Patient- and family-centred care transition interventions for adults: a systematic review and meta-analysis of RCTs. Int. J. Qual. Health Care.

[bib0014] Chaudhri S., Brown L., Hassan I., Horgan A.F. (2005). Preoperative intensive, community-based vs. traditional stoma education: a randomized, controlled trial. Dis. Colon Rectum.

[bib0015] Colwell J.C., Gray M. (2007). Evidence-based report card. Does preoperative teaching and stoma site marking affect surgical outcomes in patients undergoing ostomy surgery?. J. Wound Ostomy Cont. Nurs..

[bib0016] Covidence (2025).

[bib0017] Crawford D., Texter T., Hurt K., vanAelst R., Glaza L., Vander Laan K.J. (2012). Traditional nurse instruction versus 2 session nurse instruction plus DVD for teaching ostomy care: a multisite randomized controlled trial. J. Wound Ostomy Cont. Nurs..

[bib0018] Danielsen A.K., Burcharth J., Rosenberg J. (2013). Patient education has a positive effect in patients with a stoma: a systematic review. Colorectal Dis..

[bib0019] Danielsen A.K., Rosenberg J. (2014). Health related quality of life may increase when patients with a stoma attend patient education-a case-control study. PLoS One.

[bib0020] Danielsen A.K., Rosenberg J. (2014). Patient education after stoma creation may reduce health-care costs. Dan. Med. J..

[bib0021] Department of Health and Aged Care (2024). Stoma Appliances Scheme. https://www.health.gov.au/our-work/stoma-appliance-scheme.

[bib0022] Duque P.A., Valencia Rico C.L., Campiño Valderrama S.M., López González L.A. (2023). Effects of socio-educational interventions on the quality of life of people with a digestive ostomy. SAGE Open Nurs..

[bib0023] Faury S., Koleck M., Foucaud J., M'Bailara K., Quintard B. (2017). Patient education interventions for colorectal cancer patients with stoma: a systematic review. Patient Educ. Couns..

[bib0024] Fish D.R., Mancuso C.A., Garcia-Aguilar J.E., Lee S.W., Nash G.M., Sonoda T., Charlson M.E., Temple L.K. (2017). Readmission after ileostomy creation: retrospective review of a common and significant event. Ann. Surg..

[bib0025] Forsmo H.M., Pfeffer F., Rasdal A., Sintonen H., Körner H., Erichsen C. (2016). Pre- and postoperative stoma education and guidance within an enhanced recovery after surgery (ERAS) programme reduces length of hospital stay in colorectal surgery. Int. J. Surg..

[bib0026] García-Cabrera A.M., de la Portilla de Juan F., Navarro-Morales L., Ribera García S., Durán Ventura M.d.C., Fernández Luque I., Javier Padillo-Ruiz F. (2023). Influence of preoperative educational intervention for patients undergoing fecal ostomy surgery: a comparison cohort study. J. Wound Ostomy Cont. Nurs..

[bib0027] Gibbins S. (2011). Stoma preoperative understanding and training trial (SPOUT): trials within the trial (proof of Murphy's law). J. Stomal Ther. Aust..

[bib0028] Gonella F., Valenti A., Massucco P., Russolillo N., Mineccia M., Fontana A.P., Cucco D., Ferrero A. (2019). A novel patient-centered protocol to reduce hospital readmissions for dehydration after ileostomy. Updates Surg..

[bib0029] Grimes D.A., Schulz K.F. (2002). An overview of clinical research: the lay of the land. Lancet.

[bib0030] Haines A. (2023). https://australianstoma.com.au/wp-content/uploads/ANewBeginning.pdf.

[bib0031] Harrison J.D., Auerbach A.D., Anderson W., Fagan M., Carnie M., Hanson C., Banta J., Symczak G., Robinson E., Schnipper J., Wong C., Weiss R. (2019). Patient stakeholder engagement in research: a narrative review to describe foundational principles and best practice activities. Health Expect..

[bib0032] He D., Liang W., Yao Q., Zhao J., Liu R., Chen G., Wang H., Ye X., Huang R. (2021). The effect of stoma education class on peristomal dermatitis in colorectal cancer patients with defunctioning ileostomy-a retrospective study of 491 patients. Transl. Cancer Res..

[bib0033] Heidari-Beni F., Esmaeilian S., Yousefi F., Zarei M.R., Farahani M.A. (2022). Comparison of face-to-face education and multimedia software education on adjustment of patients with intestinal ostomy: a randomized controlled trial. J. Wound Ostomy Cont. Nurs..

[bib0034] Hughes M.J., Cunningham W., Yalamarthi S. (2020). The effect of preoperative stoma training for patients undergoing colorectal surgery in an enhanced recovery programme. Ann. R. Coll. Surg. Engl..

[bib0035] International Ostomy Association (IOA) (2007). https://qldstoma.asn.au/wp-content/uploads/IOA-Charter-of-Ostomates-Rights.pdf.

[bib0036] Iqbal A., Raza A., Huang E., Goldstein L., Hughes S.J., Tan S.A. (2017). Cost effectiveness of a novel attempt to reduce readmission after ileostomy creation. JSLS: J. Soc. Laparoendosc. Surg..

[bib0037] Ketelaers S.H.J., Ham N.V., Pelt K.A.A.J.V., Timmers T., Nieuwenhuijzen G.A.P., Rutten H.J.T., Burger J.W.A., Bloemen J.G. (2023). The development and implementation of an interactive application for new ostomy patients. Colorectal Dis..

[bib0038] Khalil H., Jia R., Moraes E.B., Munn Z., Alexander L., Peters M.D.J., Asran A., Godfrey C.M., Tricco A.C., Pollock D., Evans C. (2025). Scoping reviews and their role in identifying research priorities. J. Clin. Epidemiol..

[bib0039] Kilpatrick K., Savard I., Audet L.-A., Costanzo G., Khan M., Atallah R., Jabbour M., Zhou W., Wheeler K., Ladd E., Gray D.C., Henderson C., Spies L.A., McGrath H., Rogers M. (2024). A global perspective of advanced practice nursing research: a review of systematic reviews. PLoS One.

[bib0040] Kittinouvarat S., Charoenlar S., Aeksiriwaranon W. (2011). Development of a self-care empowerment model for patients with faecal diversion. World Counc. Enteros. Ther. J..

[bib0041] Ko H.F., Wu M.F., Lu J.Z. (2023). A randomized control study: the effectiveness of multimedia education on self-care and quality of life in patients with enterostomy. Int. Wound J..

[bib0042] Koc M.A., Akyol C., Gokmen D., Aydin D., Erkek A.B., Kuzu M.A. (2023). Effect of prehabilitation on stoma self-care, anxiety, depression, and quality of life in patients with stomas: a randomized controlled trial. Dis. Colon Rectum.

[bib0043] Levac D., Colquhoun H., O'Brien K.K. (2010). Scoping studies: advancing the methodology. Implement. Sci..

[bib0044] Lim S.H., Chan S.W.C., Chow A., Zhu L., Lai J.H., He H.-G. (2019). Pilot trial of a STOMA psychosocial intervention programme for colorectal cancer patients with stomas. J. Adv. Nurs..

[bib0045] Lin L., Fang Y., Wei Y., Huang F., Zheng J., Xiao H. (2024). The effects of a nurse-led discharge planning on the health outcomes of colorectal cancer patients with stomas: a randomized controlled trial. Int. J. Nurs. Stud..

[bib0046] Linz M.E., Xiong M., Lanser H.C., Young A.T., James M. (2025). Analysis of intestinal ostomy content on TikTok: the role of social media in countering fear and stigma. Am. J. Surg..

[bib0047] Lo S., Wang Y., Wu L., Hsu M., Chang S., Hayter M. (2010). A cost–effectiveness analysis of a multimedia learning education program for stoma patients. J. Clin. Nurs..

[bib0048] Lo S.F., Wang Y.T., Wu L.Y., Hsu M.Y., Chang S.C., Hayter M. (2011). Multimedia education programme for patients with a stoma: effectiveness evaluation. J. Adv. Nurs..

[bib0049] Morley C., Jose K., Hall S.E., Shaw K., McGowan D., Wyss M., Winzenberg T. (2024). Evidence-informed, experience-based co-design: a novel framework integrating research evidence and lived experience in priority-setting and co-design of health services. BMJ Open.

[bib0050] Özkaya E., Harputlu D. (2024). The effect of education via videoconferencing at home on individuals' self-efficacy and adaptation to life with a stoma: a randomized controlled study. Adv. Skin Wound Care.

[bib0051] Page M.J., McKenzie J.E., Bossuyt P.M., Boutron I., Hoffmann T.C., Mulrow C.D., Shamseer L., Tetzlaff J.M., Akl E.A., Brennan S.E., Chou R., Glanville J., Grimshaw J.M., Hróbjartsson A., Lalu M.M., Li T., Loder E.W., Mayo-Wilson E., McDonald S., Moher D. (2021). The PRISMA 2020 statement: an updated guideline for reporting systematic reviews. BMJ.

[bib0052] Peters M., Godfrey C., McInerney P., Munn Z., Tricco A.C., Khalil H., Aromataris E., Munn Z. (2020). JBI Reviewer's Manual.

[bib0053] Phatak U.R., Li L.T., Karanjawala B., Chang G.J., Kao L.S. (2014). Systematic review of educational interventions for ostomates. Dis. Colon Rectum.

[bib0054] Pollock D., Davies E.L., Peters M.D.J., Tricco A.C., Alexander L., McInerney P., Godfrey C.M., Khalil H., Munn Z. (2021). Undertaking a scoping review: a practical guide for nursing and midwifery students, clinicians, researchers, and academics. J. Adv. Nurs..

[bib0055] Pollock D., Peters M.D.J., Khalil H., McInerney P., Alexander L., Tricco A.C., Evans C., de Moraes É.B., Godfrey C.M., Pieper D., Saran A., Stern C., Munn Z. (2023). Recommendations for the extraction, analysis, and presentation of results in scoping reviews. JBI Evid. Synth..

[bib0056] Qiao J., Zhao Y., Lu Y., Li Q., Dong H.-J. (2024). Assessing the impact of educational eHealth and mHealth interventions on health outcomes in continuity of care for enterostomy patients: a meta-analysis. Eur. J. Oncol. Nurs..

[bib0057] Ricci L., Villegente J., Loyal D., Ayav C., Kivits J., Rat A.C. (2022). Tailored patient therapeutic educational interventions: a patient-centred communication model. Health Expect..

[bib0058] Roeung S., Lindgren T.G., Carley A. (2024). Improving discharge teaching for adult patients with an ileostomy. Am. J. Nurs..

[bib0059] Seo H.W. (2019). Effects of the frequency of ostomy management reinforcement education on self-care knowledge, self-efficacy, and ability of stoma appliance change among Korean hospitalised ostomates. Int. Wound J..

[bib0060] Shepherd T., Trinder M., Theophilus M. (2024). Does virtual reality in the preoperative setting for colorectal cancer surgery improve patient understanding? A randomized pilot study [Randomized Controlled Trial]. ANZ J. Surg..

[bib0061] Shi V., McKechnie T., Anant S., Pedroso C.M., Ahmed M., Patel J., Sharma S., Talwar G., Hong D., Eskicioglu C. (2025). The impact of preoperative stoma education on postoperative outcomes for patients with new stomas after colorectal surgery: a systematic review and meta-analysis. Tech. Coloproctol..

[bib0062] Soares-Pinto I.E., Queirós S.M.M., Silva C.R.R.D., Alves P.J.P., Santos C.S.V.D.B., Brito M.A.C.D. (2025). Nursing intervention program for intestinal stoma self-care: a focus group. Referência.

[bib0063] Song Q.F., Yin G., Guo X., Lv X., Yu K., Liu C. (2021). Effects of a self-management program for patients with colorectal cancer and a colostomy: a nonrandomized clinical trial. J. Wound Ostomy Cont. Nurs..

[bib0064] Stokes A.L., Tice S., Follett S., Paskey D., Abraham L., Bealer C., Keister H., Koltun W., Puleo F.J. (2017). Institution of a preoperative stoma education group class decreases rate of peristomal complications in new stoma patients. J. Wound Ostomy Cont. Nurs..

[bib0065] Tan S.B.T., Fung C.Y.K., Walters S., Chiam H.C., Ruggiero B., Naidoo M., Young C.J., Cheong J.Y. (2025). Factors contributing to delayed discharge related to stoma education in patients undergoing colorectal surgery: a retrospective analysis. ANZ J. Surg..

[bib0066] The Endnote Team. (2013). *Endnote*. In (Version Endnote 21) [64 bit]. Clarivate.

[bib0067] Tricco A.C., Lillie E., Zarin W., O'Brien K.K., Colquhoun H., Levac D., Moher D., Peters M.D.J., Horsley T., Weeks L., Hempel S., Akl E.A., Chang C., McGowan J., Stewart L., Hartling L., Aldcroft A., Wilson M.G., Garritty C., Straus S.E. (2018). PRISMA extension for scoping reviews (PRISMA-ScR): checklist and explanation. Ann. Intern. Med..

[bib0068] Tricco A.C., Lillie E., Zarin W., O’Brien K., Colquhoun H., Kastner M., Levac D., Ng C., Sharpe J.P., Wilson K., Kenny M., Warren R., Wilson C., Stelfox H.T., Straus S.E. (2016). A scoping review on the conduct and reporting of scoping reviews. BMC Med. Res. Methodol..

[bib0069] Tweed T.T.T., Woortman C., Tummers S., Bakens M., van Bastelaar J., Stoot J. (2021). Reducing hospital stay for colorectal surgery in ERAS setting by means of perioperative patient education of expected day of discharge. Int. J. Colorectal. Dis..

[bib0070] van Loon Y.T., Clermonts S., Belt R., Nagle D., Wasowicz D.K., Zimmerman D.D.E. (2020). Implementation of an easy in-hospital educational stoma pathway results in decrease of home nursing care services after discharge. Colorectal Dis..

[bib0071] van Pelt K.A.A.J., van Loon Y.T., Schots J.P.M., Ketelaers S.H.J., Zimmerman D.D.E., Nieuwenhuijzen G.A.P., Rutten H.J.T., Burger J.W.A., Bloemen J.G. (2024). Effects of a perioperative educational pathway on ostomy self-care, level of independence and need for visiting nurse services: a comparative observational cohort study. Colorectal Dis..

[bib0072] Wainwright T.W., Jakobsen D.H., Kehlet H. (2022). The current and future role of nurses within enhanced recovery after surgery pathways. Br. J. Nurs..

[bib0073] Wang S.Y., Chang T.H., Han C.Y. (2021). Effectiveness of a multimedia patient education intervention on improving self-care knowledge and skills in patients with colorectal cancer after enterostomy surgery: a pilot study. Adv. Skin Wound Care.

[bib0074] Wang Y., Ren H., Li M., Xie L., Lin L., Fang Y.L. (2024). Effect of enterostomal therapist-led visual health education combined with peer education on the self-nursing ability, quality of life and peristomial complications in patients with a permanent colostomy. Patient Prefer. Adherence.

[bib0075] World Health Organization (2025). https://www.who.int/health-topics/integrated-people-centered-care#tab=tab_1.

[bib0076] Yang Q., Van Stee S.K. (2019). The comparative effectiveness of mobile phone interventions in improving health outcomes: meta-analytic review. JMIR. MHealth UHealth.

[bib0077] Yeo H., Park H. (2023). Benefits of a single-session, in-hospital preoperative education program for patients undergoing ostomy surgery: a randomized controlled trial. J. Wound Ostomy Cont. Nurs..

[bib0078] Yiğitoğlu E.T., Şendir M. (2021). Effect of a mobile patient education application on adjustment to stoma and development of peristomal skin lesions: a quasi-experimental study. Wound Manage Prev..

[bib0079] Younis J., Salerno G., Fanto D., Hadjipavlou M., Chellar D., Trickett J.P. (2012). Focused preoperative patient stoma education, prior to ileostomy formation after anterior resection, contributes to a reduction in delayed discharge within the enhanced recovery programme. Int. J. Colorectal. Dis..

[bib0080] Zhou L., Zhang F., Li H., Wang L. (2023). Post-discharge health education for patients with enterostomy: a nationwide interventional study. J. Glob. Health.

