# Supplementary material for: Perioperative education for patients undergoing colorectal stoma surgery: A scoping review
Source: Int J Nurs Stud Adv. 2026 Jun 16;11:100604. doi: 10.1016/j.ijnsa.2026.100604 (PMC13312106; doi:10.1016/j.ijnsa.2026.100604)
Supplement: Supplementary file 1 [file mmc1.docx]

**Appendix I:** Final search strategy of all included databases (CINAHL, MEDLINE, PsycINFO, Scopus)

| Search engine | Keyword search terms | Number of Papers |
| --- | --- | --- |
| CINAHL | ((MH “Enterostomy+”) OR (MH “Enterostomal Therapy Nursing”) OR (MH “Wound, Ostomy and Continence Nursing+”)) AND (MH “Patient Education+”) | 517 |
| MEDLINE | (MH “Enterostomy” +) AND (MH “Patient Education as Topic+”) | 363 |
| PsycINFO | (DE “Colostomy”) AND ((DE “Client Education”) OR (DE “Health Education”)) | 3 |
| Scopus | (TITLE-ABS-KEY ("patient education" OR "client education" OR "health education" OR "perioperative education" OR "preoperative education" OR "pre-operative education")) AND (TITLE-ABS-KEY (enterostomy OR colostomy OR ileostomy OR cecostomy OR "enterostomal therapy nursing" OR "wound, ostomy and continence nursing") ) | 605 |
