# Supplementary material for: Perioperative education for patients undergoing colorectal stoma surgery: A scoping review
Source: Int J Nurs Stud Adv. 2026 Jun 16;11:100604. doi: 10.1016/j.ijnsa.2026.100604 (PMC13312106; doi:10.1016/j.ijnsa.2026.100604)
Supplement: Supplementary file 3 [file mmc3.docx]

Supplementary Table 1. Characteristics of included studies

| First author, year,  country and  study design | Aim or research question/s | Sample and type of stoma | Education intervention | Time and setting | Key findings |
| --- | --- | --- | --- | --- | --- |
| Abdelmohsen (2020)  Egypt  Analytical | To evaluate the effectiveness of structured education on patient’s knowledge and practice regarding colostomy care | N=60  Colostomy | Intervention: One 45‑minute theoretical session (booklet, lecture, group discussion) and two 45‑minute practical sessions including stoma‑care demonstration and instructional video | Postoperative: outpatient clinic | Intervention statistically improved knowledge scores and stoma care skills |
| Alenezi (2016)  Saudi Arabia  RCT | To evaluate the impact of stoma care education in minimizing stoma-related complications | N=100  Colostomy and Ileostomy | Control: did not receive the education session prior to discharge; standard care was not described  Intervention: One‑on‑one postoperative education prior to discharge; peristomal skin assessed at 1, 3, and 6 weeks | Postoperative: surgical ward | Intervention group had significantly fewer peristomal skin complications at 1, 3 and 6 weeks |
| Çakır (2018)  Türkiye  RCT | To analyse the impact of preoperative  education on the anxiety levels of patients undergoing planned colorectal surgery involving stoma formation | N=60  Colostomy and Ileostomy | Control: routine education in clinic  Intervention: in addition to routine education, the group received education preoperatively based on a training manual, viewed a DVD postoperatively, and performed their own stoma care with supervision prior to discharge. | Preoperative: clinic  Postoperative: hospital | Intervention group had statistically lower state/trait anxiety scores than the control group following the preoperative education, and at 6 months postoperatively. |
| Chaudri (2005)  United Kingdom  RCT | To compare preoperative intensive, community-based stoma education with conventional postoperative stoma education after elective colorectal surgery | N=42  Colostomy and Ileostomy | Control: one 60-minute preoperative education session receiving information on stomas and observation of stoma appliances, education on day of admission, daily postoperative education and three postoperative visits  Intervention: two 45‑minute preoperative home visits using audiovisual aids and a stoma model for practice, in addition to standard care | Preoperative: clinic and home  Postoperative: hospital and community | Statistically significant findings in the intervention group requiring less time to achieve stoma proficiency, shorter hospital stays, and fewer complications post discharge. This resulted in reduced mean cost of healthcare |
| Crawford (2012)  USA  RCT | To compare two methods of ostomy care instruction to determine their effect on patients’ knowledge, skills, and confidence related to postoperative ostomy care | N=68  Colostomy and Ileostomy | Control: three one-on-one sessions including hands-on skill training and printed material  Intervention: two one-on-one sessions plus a DVD | Postoperative: hospital | Nurse instruction plus DVD was as effective as the standard education. No significant difference between the two methods of ostomy care instruction. |
| Danielsen  (2014a)  Denmark  Non-RCT | To explore the effect of a structured patient education program on health-related Quality of Life (QoL). | N=50  Colostomy and Ileostomy | Control: preoperative routine stoma education and postoperative guidance with contact at 10 days, 1, 3, 6 and 12 months  Intervention: additional stoma‑nurse ward visit for pouch‑change guidance, phone call 5 days after discharge, and three multidisciplinary group sessions | Postoperative: hospital | A significant improvement in QoL in the intervention group between baseline, 3 and 6 months.  The control group showed no variation.  No significant differences between groups at 3- and 6-months post stoma creation |
| Duque (2023)  Colombia  RCT | To describe the effect of a socio-educational nursing intervention on the QoL of people with a digestive ostomy | N=12  Colostomy and Ileostomy | Control: single personalised theoretical session face-to-face assisted by use of video material  Intervention: personalised theoretical-practical session delivered face-to-face followed by a home visit | Postoperative: outpatient clinic and home | The QoL dimensions showed an increase in both groups without statistical difference. The physical wellness, body image and social concerns dimensions statistically improved in the control group. The body image and positive adaptation dimensions significantly improved in the intervention group |
| Forsmo (2016)  Norway  RCT | To determine whether an ERAS care pathway can reduce the total postoperative hospital stay compared to standard care | N=122  Colostomy and Ileostomy | Control: individual preoperative consultation from nurses with varying experience in stoma care on the day of admission (day before surgery); daily postoperative education by ward nurses with sporadic supervision from stoma nurse specialist  Intervention: Individual preoperative consultation with ERAS and stoma nurses including demonstration and practice, stoma equipment to take home, information brochure, and daily postoperative one-on-one education | Preoperative: clinic  Postoperative: surgical ward | Intervention resulted in significantly shorter LoS. No significant differences exhibited in major or minor morbidities, reoperation rate, readmission rate, and 30-day mortality |
| García-Cabrera  (2023)  Spain  Analytical Study | To evaluate effectiveness of a preoperative education program in the outpatient setting in reducing length of stay for ostomy patients, compared to standard postoperative care | N=138  Colostomy and Ileostomy | Control: preoperative stoma siting; postoperative education and counselling in hospital 24-48 hours after surgery  Intervention: standard care with an additional 45-minute one-on-one preoperative education and counselling session by stoma nurse | Preoperative: clinic | No difference in LoS between groups. Complications, mortality, and readmission rates significantly higher in the preoperative education group |
| Gibbins  (2011)  Australia  RCT | To demonstrate the outcomes of a novel preoperative stoma care training protocol (SPOUT) compared to conventional care for elective stoma surgery | N=14  Colostomy and Ileostomy | Control: one-on-one preoperative education by stoma nurse with verbal instruction, illustrations, information booklet  Intervention: additionally received demonstration of changing a stoma appliance, stoma appliance to wear home and remove later, a stoma care DVD, and extended booklet | Preoperative: clinic | Some correlation between intervention group and less time for postoperative education was found.  No statistically significant findings |
| Gonella  (2019)  Italy  Analytical Study | To evaluate the efficacy of a new multidisciplinary individualized multi-step protocol in terms of reduction of hospital readmission for dehydration | N=296  Ileostomy | Intervention: two one-on-one preoperative education sessions by stoma nurse; postoperative counselling by surgeon, dietician and stoma nurse with verbal instruction and printed information, and phone follow-up according to dehydration protocol | Preoperative: clinic  Postoperative: hospital and outpatient phone call | Hospital readmission rate within 30 days post discharge for dehydration reduced from 9% to 3.9%.  Number of avoided potential readmissions was 29/129 (22.4%) due to recall schedule interventions |
| He  (2021)  China  Analytical Study | To determine if well-educated patients will have a lower risk of peristomal dermatitis in the early postoperative period compared to patients who did not participate in the stoma education class | N=491  Ileostomy | Intervention: postoperative demonstration and practice of stoma appliance change prior to discharge; 1-hour post-discharge group education class with stoma nurse and volunteer ostomate support for patients and carers with slides, question and answer time, and information booklet | Postoperative: hospital and outpatient clinic | Stoma education was the only factor associated with a statistically significant decrease in peristomal dermatitis with diabetes being an independent risk factor |
| Heidari-Beni (2022)  Iran  RCT | To compare the effect of face-to-face versus multimedia education on the adjustment of patients to an intestinal ostomy | N=126  Colostomy and Ileostomy | Control: routine care with no additional education.  Intervention 1 (face to face): 3-hour sessions over four consecutive days with information booklet to share with caregivers and 3 months phone follow-up  Intervention 2 (multimedia): postoperative viewing of multimedia educational program on laptop with CD to take home and view with family/carers and 3 months phone follow-up | Postoperative: hospital, home, and outpatient phone call | Multimedia and face-to-face education significantly improved mean adjustment scores (Ostomy Adjustment Inventory-23) three months after the education. The improved scores of the multimedia group were statistically significant compared to face-to-face education and standard care. |
| Hughes (2020)  United Kingdom  Analytical Study | To assess the impact of preoperative stoma training on length of stay. Secondary outcomes measured were overall morbidity, stoma related morbidity, ERAS milestone achievement and readmission rates. | N=123  Colostomy and Ileostomy | Preoperative education and training by stoma nurse with verbal instruction, training pack with mock stoma and appliances to practice with at home | Preoperative: clinic | Preoperative education significantly reduced median length of stay. No significant difference in overall morbidity rates, stoma related morbidity or readmissions. |
| Iqbal (2017)  USA  Analytical Study | To determine the clinical and economic impact of an outpatient intervention to decrease readmissions for dehydration after ileostomy creation | N=55  Ileostomy | Postoperative one-on-one dehydration education and recording of ileostomy output; 21-day phone follow-up | Postoperative: hospital and outpatient phone call | Standard ileostomy education and phone calls postoperatively significantly reduced dehydration-related readmissions and was cost-effective |
| Ketelaers (2023)  The Netherlands  Analytical Study | To develop an interactive application (app) to educate patients scheduled for ostomy surgery and to evaluate implementation of the app (StoManager) | N=30  Colostomy and Ileostomy | Training in app use provided to patients. Patients could access app education at any time up to 10 weeks post-surgery | Preoperative:  Hospital  Postoperative: at home | The app proved feasible with high satisfaction rates from patients. At discharge, 28/30 (93.3%) patients did not require home nursing services for ostomy care |
| Kittinouvarat (2011)  Thailand  Analytical | To compare knowledge, self-care behaviour, satisfaction of self-care behaviours and study satisfaction of the faecal diversion patients before and after receiving the Self-Care Empowerment Programme (SCEP) | N=15  Colostomy and Ileostomy | Intervention: preoperative one-on-one psycho-educative programme (30-50 minutes) with verbal information, and self-care manual; two 50-minute postoperative visits in hospital with one-on-one demonstration and practice of stoma appliance change; two 50-minute outpatient clinic visits 2 weeks and 6 weeks after discharge | Preoperative: clinic  Postoperative: hospital and outpatient clinic | Preoperative and postoperative education significantly improved stoma self-care knowledge; no difference found between self-care behaviours or self-care behaviour satisfaction between the patients in week 2 and 6 after discharge; patient satisfaction after attending the programme was at an excellent level |
| Ko (2023)  Taiwan  RCT | To investigate the effectiveness of a multimedia patient education intervention on enhancing the self-care and QoL among patients with a postoperative stoma | N=108  Colostomy and Ileostomy | Control: four 3-hour individual education sessions over four consecutive days with information brochure for patient and family  Intervention: postoperative multimedia educational program on laptop computer for use by patient and family at home | Postoperative: at home | Intervention group had significantly improved self-care ability and QoL scores 3 months after the intervention than the control group |
| Koc (2023)  Türkiye  RCT | To measure the effects of prehabilitation on stoma self-care, QoL, anxiety, and depression levels | N=218  Colostomy and Ileostomy | Intervention A: received stoma siting and information on living with a stoma from stoma nurse during a face-to-face session preoperatively; education on stoma care commenced postoperative day one  Intervention B: additionally received a 45-minute face-to-face education session on stoma care preoperatively as well as on postoperative day one  Intervention C: additionally received a water-filled ostomy appliance attached 48hours before surgery and education on managing the appliance by the stoma nurse. The appliance was not removed until surgery. | Preoperative: clinic and hospital  Postoperative: in hospital | Intervention Group C had significantly higher QoL and self-care than other groups |
| Lim (2019)  Singapore  RCT | To evaluate the preliminary effects of a STOMA psychosocial intervention programme on the outcomes of colorectal cancer patients with a newly formed stoma | N=51  Colostomy and Ileostomy | Control: received stoma siting, stoma education commenced on day 3-5 postoperatively with demonstration and practice of stoma appliance change. Patients return post discharge for further education if required  Intervention: additionally receives preoperative one-on-one education; educational booklet; one follow-up phone call preoperatively and four follow-up phone calls postoperatively by experienced nurse | Preoperative: clinic and phone call  Postoperative: hospital and outpatient phone call | Intervention group had significantly improved stoma acceptance amongst patients. There were no significant effects on stoma-care efficacy, stoma proficiency, length of stay, anxiety and depression level or QoL. |
| Lin (2024)  China  RCT | To evaluate the effects of nurse-led discharge planning on the quality of discharge education, stoma self-efficacy, readiness for hospital discharge, stoma QoL, incidence of stoma complications, unplanned re-admission rate, and length of stay | N=153  Colostomy and Ileostomy | Control: received usual admission education, standard surgical nursing, discharge guidance before discharge, phone follow-up one week after discharge and outpatient follow-up one month after discharge by the same nurses as the intervention group  Intervention: additionally received nurse‑led discharge planning starting within 24 hours of admission; postoperative one-on-one verbal education supported with a manual, stoma video, stoma model, stoma products; self-management sheet, discharge standards sheet, medication list, discharge referrals, outpatient follow-up, post-discharge phone and We-chat messaging follow-up | Postoperative: hospital, outpatient clinic phone call, and messaging | Nurse-led discharge planning in addition to usual discharge education showed significantly improved quality of discharge teaching, readiness for hospital discharge, stoma self-efficacy, stoma QoL, complications, and unplanned readmissions |
| Lo (2010)  Taiwan  RCT | To compare the costs and effectiveness of enterostomal education using a multimedia  learning education program (MLEP) and a conventional education service program (CESP) | N=54  Colostomy and  Ileostomy | Control: received standard care brochure on postoperative day one with one-on-one question and answer time with researcher  Intervention: additionally viewed multimedia (film and pictures)  education on day one postoperatively | Postoperative: in hospital | Participants showed significantly better outcomes after one week in the measures of: knowledge of self-care, attitude of self-care, behaviour of self-care. The cost effectiveness showed the MLEP model was better than the CESP |
| Lo (2011)  Taiwan  RCT | To evaluate the effectiveness of a multimedia education program in relation to stoma knowledge, self-care attitudes and behaviour with patients with a stoma in the postoperative period | N=102  Colostomy and  Ileostomy | Control: received standard care brochure on postoperative day one with one-on-one question and answer time with researcher  Intervention: additionally viewed multimedia (film and pictures)  education on day one postoperatively | Postoperative: in hospital | Multimedia education significantly improved self-care knowledge, self-care attitudes, and self-care behaviour after 1 week |
| Özkaya (2004)  Türkiye  RCT | To determine the effect of post-discharge stoma care education provided at home via videoconference on individuals’ self-efficacy and adaptation to life with a stoma | N=60  Colostomy and  Ileostomy | Control: received face-to-face preoperative education with an information booklet according to type of stoma  Intervention: additionally received four postoperative one-to-one education sessions at home via video calls | Preoperative: clinic  Postoperative: outpatient video call | Intervention group had significantly improved stoma self-efficacy and ostomy adjustment scores compared to the control group |
| Seo (2018)  South Korea  RCT | To evaluate the effects of ostomy management reinforcement education (OMRE) on self-care knowledge, self-efficacy, and ability to change stoma appliance in ostomates and to identify the optimal frequency of reinforcement education | N=60  Colostomy and  Ileostomy | Control: OMRE provided once  Intervention 1: OMRE provided twice  Intervention 2: OMRE provided three times Education provided one-on-one with additional self-care education, demonstration and practice changing the ostomy appliance | Postoperative: in hospital | The addition of two postoperative education sessions to the standard education significantly improved self-care knowledge, self-efficacy, and ability of stoma appliance change |
| Song (2021)  China  Non-RCT | To construct a multimedia messaging app platform, Chronic Self-Management Program, and assess its effects on self-efficacy, self-management ability, and stomal and peristomal complications of patients with colorectal cancer and a colostomy | N=85  Colostomy and Ileostomy | Control: received education and counselling on ostomy care and daily life with an ostomy, a stomal care manual prior to discharge, and follow-up phone calls twice a month for 3 months  Intervention: additionally received access to a multimedia messaging app for 6 weeks post discharge with daily informational messages, weekly discussion forums and private messaging support from psychological counsellors and wound, ostomy and continence nurses | Postoperative:  hospital, outpatient phone call and messaging | Intervention group reported significantly higher self-efficacy scores, significantly improved self-management ability and significantly lower incidence of peristomal complications 6 weeks post intervention compared to the control group |
| Stokes (2017)  USA  Analytical Study | To compare selected postoperative complications, hospital length of stay, and readmission rates in a group of patients who attended a preoperative educational intervention to a retrospective group of patients who did not receive the intervention | N=218  Colostomy and Ileostomy | Intervention: participants attended a 2-hour preoperative group education session about the surgery and managing stomas, with a demonstration, and practice of changing an ostomy appliance | Preoperative: clinic | Patients who attended the 2-hour education class had significantly fewer peristomal complications compared to the retrospective group of patients. There were no significant differences in length of stay or 30-day readmission rate |
| Tweed (2024)  The Netherlands  Analytical Study | To investigate reduction of length of stay by perioperative education with an expected discharge date. Secondary outcomes measured were readmission, complications and 90-day mortality. | N=578  Colostomy and Ileostomy | Historical control group: (ERAS): received ERAS standard care with an ERAS information booklet of preoperative, perioperative, and postoperative procedures; postoperative guidance from an ERAS nurse  Intervention: (ERAS+): additionally received two preoperative one-on-one education of discharge date and discharge criteria; and daily confirmation of expected discharge date | Preoperative: clinic and hospital  Postoperative: hospital | The ERAS+ group had a significantly shorter median length of stay compared to patients in the ERAS group. No statistical difference was found in the number of readmissions, complications or mortality between the two groups |
| van Pelt (2024)  The Netherlands  Analytical Study | To determine if a perioperative ostomy educational pathway increases the level of independence and decreases the need for visiting nurse services in new ostomy patients | N=277  Colostomy and Ileostomy | Historical control group: did not attend preoperative practice session  Intervention: preoperative one-on-one education and practice with ostomy appliance, postoperative practice emptying and changing appliance over 5 days in hospital with assistance of nurse or stoma nurse | Preoperative: clinic | After discharge, the intervention group were significantly more independent performing ostomy care and were significantly less reliant on community nurses |
| Wang (2021)  Taiwan  Non-RCT | To examine the effects of a multimedia patient education  intervention on improving self-care knowledge and skills in patients with colorectal cancer who underwent colostomy surgery | N=63  Colostomy | Control: received one preoperative education session with standard written and verbal nursing instructions and one postoperative education session  Intervention: received additional 10-minute multimedia DVD education that could be viewed as often as needed while in hospital | Preoperative: clinic  Postoperative: in hospital | The intervention group had significantly improved self-care knowledge and self-care skills after discharge compared to the control group |
| Wang (2024)  China  RCT | To analyse the impact of enterostomal therapist-led visual health education combined with peer education on the postoperative self-nursing ability, QoL and peristomal complications in patients with a permanent  colostomy. | N=120  Colostomy | Control: Routine care and education procedure not explained. Informed of stoma precautions by nurse prior to discharge. Patients returned to visit stoma nurse for education after discharge  Intervention: received enterostomal therapist-led visual education with video demonstration, posters, and brochures on stoma care; post-discharge weekly communication with peer-educator; and monthly group discussions with peer educator for three months post discharge | Postoperative: hospital and outpatient clinic | The intervention group had significantly improved QoL, self-nursing ability and adaptability as well as significantly lower incidence of complications and significantly higher nursing satisfaction |
| Yeo (2023)  South Korea  RCT | To evaluate the effects of a single session video-based preoperative ostomy education session on self-care knowledge, self-care proficiency, anxiety and depression, length of hospital stay, and ostomy-related complications in patients undergoing ostomy surgery | N=41  Colostomy and Ileostomy | Control: received stoma site marking and shown an ostomy appliance preoperatively. No preoperative education. Postoperatively, they received two individual education sessions to practice ostomy care and one group education session to review ostomy care and precautions  Intervention: additionally received a single 45-minute session including a video, verbal education and demonstration, practice with the stoma appliance, and question and answer time | Preoperative: clinic  Postoperative: hospital | The intervention group showed statistically improved self-care knowledge, self-care proficiency, decreased anxiety and depression scores, reduced hospital length of stay, and reduced ostomy-related complications |
| Yiğitoğlu (2021)  Türkiye  Non-RCT | To develop and evaluate an educational mobile app for stoma patients on psychosocial adjustment, self-care, and peristomal skin complications | N=60  Colostomy and Ileostomy | Control: information provided as printed material  Intervention: postoperative one-to-one verbal instruction and education on stoma care; introduction of STOMA-M mobile app prior to discharge and commenced post discharge | Postoperative: hospital and home | Individuals using the STOMA-M mobile app had statistically higher levels of adjustment to stoma, ability to self-care for their stoma, and satisfaction with education in the third month. There were no significant improvements in the prevention of peristomal skin lesions |
| Younis (2012)  United Kingdom  Non-RCT | To compare whether the hospital stay for patients with defunctioning loop ileostomies following anterior resection was prolonged due to delayed stoma management before and after the introduction of an enhanced recovery program (ERP) with improved preoperative patient stoma education | N=240  Ileostomy | Control: pre-ERP group received preoperative one-to-one education and counselling and postoperative stoma support and training by nursing staff in the hospital and community  Intervention: post-ERP group additionally received preoperative DVD demonstration of ostomy appliance emptying and changing, and a practise pack containing a self-adhesive foam stoma and ostomy appliance to practise with prior to admission | Preoperative: clinic and home  Postoperative: in hospital and community | Average length of stay reduced for ERP patients.  Postponed discharge due to a delay in independent stoma management was significantly reduced with the introduction of ERP and stoma management education |
| Zhou (2023)  China  Analytical | To understand the impact of health education based on WeChat health management program on the psychosocial level and self-ostomy care ability of stoma patients after discharge | N=4201  Colostomy and Ileostomy | Intervention: WeChat app‑based education delivered at weeks 1, 3, 7, 11, and 23 post‑discharge with online/phone support | Postoperative: day of and post discharge | Self-care ability and psychological adaptation scores were significantly higher after the intervention |

Key: ERAS: Enhanced Recovery After Surgery; GP: General Practitioner; LoS: Length of Stay; Non-RCT: Non-Randomised Controlled Trial; QoL: Quality of Life; RCT: Randomised Controlled Trial; App: Application
